# Evaluations of Actiheart, IDEEA® and RT3 monitors for estimating activity energy expenditure in free-living women

**DOI:** 10.1017/jns.2013.18

**Published:** 2013-09-06

**Authors:** Marie Löf, Hanna Henriksson, Elisabet Forsum

**Affiliations:** 1 Department of Clinical and Experimental Medicine, Faculty of Health Science, Linköping University, Linköping, Sweden; 2 Department of Biosciences and Nutrition, Karolinska Institutet, Stockholm, Sweden

**Keywords:** Activity energy expenditure, Accuracy, Activity monitors, Doubly labelled water, AEE, activity energy expenditure, AEE5dresult, total energy expenditure, measured using the doubly labelled water method during days 1–5 minus BMR measured using indirect calorimetry, AEEActi, activity energy expenditure assessed using Actiheart, AEEIDEEA, activity energy expenditure assessed using IDEEA, AEEref, activity energy expenditure assessed using the doubly labelled water method and indirect calorimetry, AEERT3, activity energy expenditure assessed using RT3, CountsActi, counts using Actiheart, CountsIDEEA, counts using IDEEA, CountsRT3, counts using RT3, DIT, dietary induced thermogenesis, HRaR, heart rate above resting heart rate, MET, metabolic equivalent, TEE, total energy expenditure, TEE5dresult, TEE during days 1–5, TEEIDEEA, total energy expenditure measured using IDEEA, TEEref, total energy expenditure measured using the doubly labelled water method

## Abstract

Activity energy expenditure (AEE) during free-living conditions can be assessed using devices based on different principles. To make proper comparisons of different devices' capacities to assess AEE, they should be evaluated in the same population. Thus, in the present study we evaluated, in the same group of subjects, the ability of three devices to assess AEE in groups and individuals during free-living conditions. In twenty women, AEE was assessed using RT3 (three-axial accelerometry) (AEE_RT3_), Actiheart (a combination of heart rate and accelerometry) (AEE_Acti_) and IDEEA (a multi-accelerometer system) (AEE_IDEEA_). Reference AEE (AEE_ref_) was assessed using the doubly labelled water method and indirect calorimetry. Average AEE_Acti_ was 5760 kJ per 24 h and not significantly different from AEE_ref_ (5020 kJ per 24 h). On average, AEE_RT3_ and AEE_IDEEA_ were 2010 and 1750 kJ per 24 h lower than AEE_ref_, respectively (*P* < 0·001). The limits of agreement (± 2 sd) were 2940 (Actiheart), 1820 (RT3) and 2650 (IDEEA) kJ per 24 h. The variance for AEE_RT3_ was lower than for AEE_Acti_ (*P* = 0·006). The RT3 classified 60 % of the women in the correct activity category while the corresponding value for IDEEA and Actiheart was 30 %. In conclusion, the Actiheart may be useful for groups and the RT3 for individuals while the IDEEA requires further development. The results are likely to be relevant for a large proportion of Western women of reproductive age and demonstrate that the procedure selected to assess physical activity can greatly influence the possibilities to uncover important aspects regarding interactions between physical activity, diet and health.

During recent decades lifestyle-related health problems have become common worldwide, important reasons being poor dietary habits with a high intake of energy and lack of physical activity^(^^1^^–^^3^^)^. The beneficial effects of physical activity on human health are due, to a large extent, to its ability to increase energy metabolism. Furthermore, physical activity is defined as muscular activity that increases energy expenditure^(^^4^^)^. Therefore, procedures to assess physical activity should be evaluated using methods able to measure energy expenditure in response to physical activity. This can be achieved by using the doubly labelled water method to assess total energy expenditure (TEE) during free-living conditions and indirect calorimetry to assess BMR, a combination referred to as ‘reference methods’ in the following. This approach makes it possible to calculate activity energy expenditure (AEE) as TEE minus BMR and provides the average amount of energy expended in response to physical activity during the study period.

Questionnaires may be used to assess physical activity^(^^5^^)^ since they are comparatively cheap and easy to use but are influenced by subjective factors such as the capacity of individuals to report their activity accurately. Neilson *et al.*^(^^5^^)^ reviewed studies evaluating such questionnaires and found that their validity often was poor at the group as well as at the individual level. Thus, there is a need for objective methods to assess physical activity. Currently used methods of this kind are based on different principles, for example, heart rate monitoring, an old and well-established technique^(^^6^^)^, movement registration, or combinations of these principles^(^^7^^)^. Such a combination is used in the Actiheart monitor (CamNtech Ltd), which has been applied in studies to assess AEE in pregnant^(^^8^^)^ and obese^(^^9^^,^^10^^)^ subjects. Actiheart is used in several on-going cohort studies investigating variations in physical activity between individuals in relation to health^(^^11^^)^. Monitors recording body movements in one axis were developed several years ago and later monitors recording body movements in several axes became available. There are a number of such monitors^(^^12^^)^, for example, the RT3 (Stayhealthy Inc.), a three-axial accelerometer. A different kind of movement registration is used by the IDEEA^®^ (Intelligent Device for Energy Expenditure and Physical Activity; MiniSun LLC) device. This system identifies the amount of time spent in different activities using sensors attached to different parts of the body^(^^13^^,^^14^^)^. This principle is interesting since it is different from principles used in other accelerometers. The potential of this system may be considerable but its validity is insufficiently known and it may need further development before it can be applied in studies.

There are a large number of studies reporting the potential of different devices to assess physical activity as evaluated by means of reference methods^(^^15^^–^^20^^)^. In a review from 2007, Plasqui & Westerterp^(^^17^^)^ concluded that three-axial accelerometers produced more accurate results than did those recording movements in one dimension only, while the great variability in the validity of different accelerometers to assess physical activity is emphasised in a more recent review by Plasqui *et al.*^(^^12^^)^. Another review by Van Remoortel *et al.*^(^^19^^)^ found evidence indicating that tri-axial accelerometers as well as multisensory monitors (including the Actiheart and the IDEEA) tend to be superior to uniaxial accelerometers^(^^19^^)^. However, the conclusions from these reviews^(^^12^^,^^17^^,^^19^^)^ are based on comparisons of data obtained in many groups of subjects differing considerably with respect to factors such as age, sex, body composition, health status and pattern of physical activity. It is very likely that the performance of monitors is influenced by such factors and therefore it is necessary to compare the capacity of uniaxial and tri-axial accelerometers to assess physical activity simultaneously in the same group of subjects. Such a comparison has been performed for Actiheart and RT3 in lean and obese men^(^^21^^)^. The results indicated that Actiheart was superior to RT3. In the present paper we use reference methods to evaluate the capacity of three different devices, the Actiheart, the IDEEA and the RT3, to assess AEE during free-living conditions. The devices were evaluated simultaneously in one group of healthy women.

## Subjects and methods

### Comments on design

Actiheart and RT3 provide estimates of AEE while IDEEA estimates TEE. The manufacturer of the IDEEA provided guidance regarding how to calculate AEE from the TEE values produced by the monitor. Therefore AEE was considered to be the appropriate estimate in the present study. However, reference methods can only produce estimates of AEE with the so-called dietary induced thermogenesis (DIT) included. In current evaluations of devices intended to assess energy expenditure in response to physical activity it is common to calculate AEE as 0·9 × TEE minus BMR, thus assuming that DIT represents 10 % of TEE. Therefore, it was important to determine if the estimates of AEE produced by the monitors included DIT. For reasons given below, we decided to compare AEE, as obtained with the three monitors, with TEE minus BMR with no deduction for DIT. Furthermore, unfortunately, the manufacturers of all three monitors provided only incomplete information regarding data acquisition and processing. Therefore we were careful to follow the recommended procedures and all questions we had during the study were resolved after discussion with a commercial representative.

### Study outline

Each woman collected two to three urine samples at home and brought them to the measurement session at the hospital, which started with a measurement of BMR. The woman's heart rate was measured during the BMR measurement by means of the Actiheart. Subsequently, after a standardised breakfast (42 kJ/kg fat-free mass and 15 % of total energy from protein), the woman performed seven standardised activities with the Actiheart attached to her body. Her energy expenditure was measured simultaneously using indirect calorimetry to establish equations producing estimates of AEE appropriate for each individual woman as requested when the Actiheart is applied. Details of this procedure are given below. The woman was then given a dose of doubly labelled water, and asked to collect six urine samples during the subsequent 14 d (days 1–15) to measure her TEE during days 1–5 (TEE_5dresult_) as well as during days 1–14 (TEE_ref_). The urine samples were to be taken in the morning on days 1, 4, 6, 8, 11 and 15 and the woman was asked to carefully note the time of sampling. Before leaving the hospital the three monitors were attached to the woman's body and she was asked to wear the Actiheart and RT3 until day 15 and the IDEEA until day 6. The purpose was to record counts (Counts_Acti_), heart rate and AEE (AEE_Acti_) by means of the Actiheart; counts (Counts_IDEEA_) and AEE (AEE_IDEEA_) by means of the IDEEA; and counts (Counts_RT3_) and AEE (AEE_RT3_) by means of the RT3. The woman was instructed that she should always wear the monitors, except when in water or sleeping, and to record in a notebook when they were taken off, as well as the activities then performed (for example, showering or sleeping). She was also instructed to attach all monitors at the same time in the morning and to remove them simultaneously just before going to bed. After the 14 d period the women returned to the hospital to deliver urine samples, monitors and the notebook. The present study was conducted according to the guidelines laid down in the Declaration of Helsinki and all procedures involving human subjects were approved by the Central Ethical Review Board, Stockholm, Sweden. Verbal informed consent, witnessed and formally recorded, was obtained from all women.

### Sample size and subjects

The initial evaluation of the monitors was conducted by means of the Bland–Altman procedure^(^^22^^)^ which has the capacity to provide descriptive and relevant information of different methods. The original description of this procedure is based on one example with seventeen observations^(^^22^^)^. The information thus obtained may motivate subsequent evaluations including testing of specific hypothesis. When planning the present study we wanted to identify differences in average AEE values related to a possible practical application of the monitors. We also considered available information regarding the principles used by the monitors to record physical activity. In particular we considered the need to assess energy expenditure to validate energy intake in dietary studies since under-reporting is a common problem in such studies^(^^23^^)^. For this application valid estimates of TEE at the group level are important and therefore a possibility to obtain valid estimates of AEE would be of value. Such estimates of AEE could then be added to BMR to obtain TEE which should be equal to the energy intake and therefore useful when evaluating dietary energy intake data. We considered that BMR can be measured without any average bias and that a bias in AEE of less than 15 % would be acceptable. This represents a bias of less than approximately 7 % in TEE since AEE is less than 50 % of TEE. In this context it may be of interest to note that Neilson *et al.*^(^^5^^)^ considered that a bias of 10 % in TEE represented acceptable criterion validity when evaluating physical activity questionnaires. We also considered that AEE_Acti_ could be expected to be identical to AEE_ref_ at the group level since a calibration, bringing the output in agreement with physiological values, is included when Actiheart is applied. With respect to AEE_IDEEA_ and AEE_RT3_ we did not know if any bias was to be expected. We assumed that AEE_ref_ was on average 5000 (sd 1000) kJ per 24 h and that the correlations between AEE_ref_ and AEE_Acti_, AEE_RT3_ or AEE_IDEEA_, respectively, were 0·5. Thus we would be able to identify a 15 % bias in AEE with a power of 0·82 with twenty subjects. Therefore twenty healthy non-smoking, non-pregnant, non-lactating women were recruited by means of advertisements in the local press in Linköping, Sweden, during the period 2007–2008. Data on their energy metabolism have been reported previously^(^^24^^,^^25^^)^.

### Activity energy expenditure assessed using reference methods

CO_2_ production and O_2_ consumption were measured during a period of 20 min after an overnight fast and 45 min of rest using the Deltratrac Metabolic Monitor (Datex Instrumentarium Corp.), and converted to BMR^(^^26^^)^. Each woman was given an accurately weighed dose of stable isotopes (0·09 g ^2^H_2_O and 0·23 g H_2_^18^O per kg body weight). Isotopic enrichments of dose and urine samples were analysed as previously described^(^^27^^)^. Analytical precision for results in parts per million was 0·22 for ^2^H and 0·03 for ^18^O. Total body water was calculated as the average of ^2^H dilution space/1·04 and ^18^O dilution space/1·01. CO_2_ production was calculated assuming 30 % of water losses to be fractionated^(^^28^^)^. TEE was calculated from CO_2_ production, assuming a food quotient of 0·85^(^^29^^)^. The ratio between ^2^H and ^18^O dilution spaces was 1·033 (sd 0·006) (*n* 20). When dose and urine samples from one subject were analysed nine times, the following CV were obtained: TEE (1·2 %), total body water (0·3 %) and fractional turnover rate constants (0·3 % or less), all well within the recommended criteria^(^^30^^)^. Reference AEE was TEE_ref_ minus BMR (AEE_ref_). Since the IDEEA was applied during 5 d only, TEE_5dresult_ minus BMR was also calculated (AEE_5dresult_).

### Actiheart

#### Monitor

The Actiheart (CamNtech Ltd; http://www.camntech.com) consists of a uniaxial accelerometer, which records bodily movements and transfers this information into counts per min, and a heart rate recorder. The device delivers information regarding AEE based on the recorded information, subject-specific information (weight, height, age and sex) and on information obtained during calibration as described above and below. According to guidance provided by the manufacturer calibration was conducted with subjects in the fed state to provide estimates of AEE with DIT included (T. Evans, CamNtech Ltd, personal communication). This procedure was selected to obtain estimates of AEE comparable with those obtained by means of IDEEA and RT3 as mentioned above. The Actiheart is attached to the chest using electrocardiography pads (2660-3; 3M Svenska AB) connecting two electrodes to the device. Actiheart software (version 4.0.11; CamNtech Ltd) was used to initiate, transfer and analyse the recorded information. As recommended by the manufacturer, before analysing the recordings, the Actiheart software was used to clean and recover or interpolate noisy and missing heart rate data for gaps less than 5 min using a built-in algorithm (www.camntech.com). We also manually investigated the heart rate recording for gaps more than 5 min and found such gaps for fourteen women. However, these gaps were few and represented only 0·26 (sd 0·22) % of the total recorded time.

#### Counts using Actiheart (Counts_Acti_) and heart rates

The number of recorded days were 14, 13, 12 and 9 for fourteen, two, two and two women, respectively. Recordings during these days covered 97 (sd 2) % of all time in the waking state. For each woman, counts assessed during all recorded days were summarised and divided by the number of recorded days to obtain Counts_Acti_ (per 24 h). For each woman and for each minute during the recorded days, heart rate above resting heart rate (HRaR) was calculated as measured heart rate minus mean resting heart rate. Subsequently, all HRaR were summarised and divided by the number of recorded minutes to obtain mean HRaR (beats per min). Resting heart rate was the average heart rate recorded when measuring BMR.

#### Calibration

All women in the study participated in the following procedure. With the Actiheart attached to her body and a nose clip to her nose, the woman was connected to a spirometer (CPX/D; Spiropharma) through a mouthpiece. Her CO_2_ production and O_2_ consumption were measured every 15 s while heart rate and counts were recorded every 1 min using Actiheart and while the woman simultaneously performed seven standardised activities (sitting, standing, walking at 3·2 km/h and 5·6 km/h, running at 8 km/h and cycling at 30 and 60 W) for 6 min each. Values recorded after 4 min were used for calculations. Energy expenditure was calculated using Weir's equation^(^^26^^)^. In this way regression lines relating heart rate and counts, respectively, to energy expenditure were established for all women in the study. All activities were used to establish regression lines for heart rate, while only resting (i.e. BMR), walking and running were used when such lines were based on counts, since the accelerometer in the Actiheart cannot distinguish between sitting, standing and cycling. For our twenty women, the correlation coefficient for heart rate *v.* energy expenditure was 0·97 (sd 0·02), while the corresponding value for counts was 0·97 (sd 0·03).

#### Calculation of activity energy expenditure assessed using Actiheart (AEE_Acti_)

For each woman, heart rates and counts assessed during the recorded days were converted to AEE based on her particular regression lines and the appropriate subject-specific information using the branched equation for heart rates and counts in the Actiheart software. AEE when not wearing Actiheart was calculated using information in the notebook as BMR of the woman times a metabolic equivalent (MET) value, appropriate for each reported activity^(^^31^^)^, times the reported duration of each particular activity minus BMR of the woman during the corresponding period of time. The amount of energy thus obtained was added to AEE, calculated using the Actiheart software as described above. This value was divided by the number of recorded days to obtain AEE_Acti_ (in kJ per 24 h). A corresponding calculation was conducted using data collected during the first 5 d of the 14 d period to obtain an AEE value comparable with AEE_IDEEA_.

### IDEEA

#### Monitor

The IDEEA device (Minisun LLC; http://www.minisun.com) consists of a microprocessor/storage unit, attached to the waist, and five sensors connected with wires to the microprocessor unit and attached to the front of the thighs, the soles of the feet and the sternum. The sensors send information regarding accelerations in two orthogonal directions and regarding angles of body parts to the microprocessor unit for identification of the following activities: lying down, reclining, transition, sitting, standing, walking, using stairs and running, and for recording the amount of time during which each activity is maintained. The device provides information, in counts per min, reflecting movements and positions of the body and combines the recorded information with subject-specific information (weight, height, age and sex) to calculate energy expenditure^(^^13^^)^ during all the time the woman wears the device. Initiation, calibration, recording and data analysis were conducted according to the manufacturer (http://www.minisun.com and M. Sun, MiniSun LLC, personal communication). Since the battery capacity of the IDEEA is only 48 h, the women were instructed to change batteries twice during the study period and all women managed to do so. The IDEEA memory capacity is limited, restricting the recording period to 5 d. During our evaluation we compared AEE_IDEEA_ with AEE_ref_, as 2 weeks is a more optimal metabolic period than 5 d for the doubly labelled water method^(^^32^^)^.

#### Counts_IDEEA_

Recordings were obtained during 3, 4 and 5 d for two, four and fourteen women, respectively, covering 98 (sd 2) % of all time in the waking state during these days. For each woman, all counts assessed during the recorded days were summarised and divided by the number of such days to obtain Counts_IDEEA_ (per 24 h).

#### Calculation of activity energy expenditure assessed using IDEEA (AEE_IDEEA_)

For each woman, energy expenditure during all activities performed when the IDEEA was worn was summarised. Energy expenditure when not wearing the IDEEA was calculated, using information in the notebook, as the resting energy metabolism (see below) of the woman times a MET value, appropriate for each reported activity^(^^31^^)^, times the reported duration of each activity. The amount of energy thus obtained was added to the energy expenditure assessed using the IDEEA as described above. In this way, TEE assessed by means of the IDEEA (TEE_IDEEA_) was obtained. TEE_IDEEA_ was divided by the number of recorded days to obtain TEE_IDEEA_ in kJ per 24 h. AEE_IDEEA_ (in kJ per 24 h) was calculated as TEE_IDEEA_ minus an estimate of the resting energy expenditure. According to a recommendation by the manufacturer, average energy expenditure when lying down during the recorded days was considered to represent the resting energy expenditure during these calculations (M. Sun, MiniSun LLC, personal communication). The equations used to predict energy expenditure by means of the IDEEA were found to be accurate for subjects in the fed state^(^^13^^)^ and thus we considered that AEE_IDEEA_ included DIT.

### RT3

#### Monitor

The RT3 (Stayhealthy Inc.; http://www.stayhealthy.com), which records movements of the body in three axes, was attached to the right hip by means of a clip. The recorded information is delivered as counts per min and is transformed into AEE (in kJ per 24 h) taking subject-specific information (weight, height, age and sex) into account. The software Stayhealthy RT3 Assist Version 1.0.7 (Stayhealthy Inc.) was used to initiate the device and to process the recorded information.

#### Counts_RT3_

All women wore the RT3 for 14 d, when the recordings covered 97 (sd 2) % of all time in the waking state. For each woman, all counts recorded during the 14 d period were summarised and divided by 14 to obtain Counts_RT3_ (per 24 h).

#### Calculations of activity energy expenditure assessed using RT3 (AEE_RT3_)

For each woman, AEE during all the time when RT3 was worn was calculated by means of the software. Energy expenditure when not wearing the device was calculated using information in the notebook as BMR times a MET value, appropriate for each reported activity^(^^31^^)^, times the reported duration of each particular activity minus the BMR during the corresponding period of time. The amount of energy thus obtained was added to AEE, calculated using the software as described above. The value obtained was divided by 14 to obtain AEE_RT3_ (in kJ per 24 h). A corresponding calculation was conducted using data collected during the first 5 d of the 14 d period to obtain an AEE value comparable with AEE_IDEEA_. The equations used to predict AEE using the RT3 software were developed with subjects in the fed state (J. Collins, Stayhealthy Inc., personal communication). Therefore we considered that AEE_RT3_ included DIT.

### Evaluation of classification capacity

This procedure evaluates the capacity of a device to rank estimates of the women in the study and therefore provides an indication of the validity of the monitor at the individual level. The procedure involves ranking women on the basis of, for example, their AEE_ref_ in a sequence. Thus, the woman with the lowest AEE_ref_ had the lowest number and the difference in AEE_ref_ between this woman and the second in the sequence was the smallest possible. This principle of the smallest possible difference was maintained for all women, producing a sequence with gradually increasing AEE_ref_. Then the women were divided into three groups with increasing AEE_ref_ comprising six (lowest), seven (middle) and seven (highest) women, respectively. This ranking and grouping procedure was carried out for AEE_Acti_, AEE_RT3_, AEE_IDEEA_, AEE_ref_, Counts_Acti_, Counts_IDEEA_, Counts_RT3_ (all expressed as per 24 h) and for HRaR (beats/min). The classification capacity was then evaluated as the number of women placed in the same (0), in the next higher (+1) or lower (–1) and in the second next higher (+2) or lower (–2) group when compared with the groups obtained when classification was based on AEE_ref_.

### Statistics

AEE assessed using each of the different monitors was compared with AEE_ref_ using the procedure described by Bland & Altman^(^^22^^)^. According to this, the difference between AEE, obtained using a monitor (AEE_Acti_, AEE_RT3_ or AEE_IDEEA_), and AEE_ref_ (*y*) was plotted against the average of the same two estimates (*x*) for all subjects. The mean difference and limits of agreement (± 2 sd) were calculated. The mean difference provides an estimate of the validity of the monitor for groups, while the limits of agreement show this validity for individual subjects. Significant differences between mean values were identified using repeated-measures ANOVA with subsequent *post hoc* analysis using Tukey's multiple-comparison test. Pearson correlation and linear regression were also used. Variances obtained for AEE_Acti_, AEE_RT3_ or AEE_IDEEA_ were compared using the *t* test for correlated variables as described by Pitman^(^^33^^)^. Significance was accepted at the *P* < 0·05 level. Values are given as means and standard deviations. All statistical analyses were conducted using Statistica software, version 8.0 (Statsoft; Scandinavia AB).

## Results

### Women and energy expenditure results

Characteristics of the women are shown in Table 1. AEE_ref_, in kJ per 24 h, was not significantly correlated with body weight (*r* 0·33; *P* = 0·16). Table 2 shows AEE_ref_ and AEE_5dresult_. On average, AEE_5dresult_ was 2 % higher than AEE_ref_ and the sd values of these two estimates were quite similar.

**Table 1.** Characteristics of the twenty women in the study (Mean values, standard deviations and ranges)

	Mean	sd	Range
Age (years)	36	8	22–45
Body weight (kg)	67·3	13·9	47·1–101·6
Height (m)	1·69	0·06	1·55–1·81
BMI (kg/m^2^)*	23·4	4·1	17·7–33·6
TEE_ref_ (kJ per 24 h)	10 930	1410	8120–13 120
TEE_5dresult_ (kJ per 24 h)	11 060	1400	8350–12 810
BMR (kJ per 24 h)	5920	740	4920–7650
PAL_ref_	1·85	0·13	1·65–2·11

TEE_ref_, total energy expenditure measured using the doubly labelled water method during days 1–15; TEE_5dresult_, total energy expenditure measured using the doubly labelled water method during days 1–5; BMR, BMR measured using indirect calorimetry; PAL_ref_, physical activity level calculated as TEE_ref_ divided by BMR.

* Three women (15 %) were overweight (BMI 25–29·9 kg/m^2^), while two women (10 %) were obese (BMI ≥ 30 kg/m^2^).

**Table 2.** Activity energy expenditure (AEE) assessed by means of the Actiheart, IDEEA and RT3 as well as by means of reference methods (*n* 20) (Mean values and standard deviations)

	AEE (kJ per 24 h)		AEE (kJ per 24 h per kg)	
	Mean	sd	Mean	sd
AEE_Acti_	5760	1380	88	25
AEE_IDEEA_	3270*†	1180	50*†	20
AEE_RT3_	3010*†	810	46*†	13
AEE_ref_	5020	890	76	15
AEE_5dresult_	5140	920	78	17

AEE_Acti,_ AEE obtained by means of the Actiheart; AEE_IDEEA,_ AEE obtained by means of the Intelligent Device for Energy Expenditure and Physical Activity (IDEEA); AEE_RT3_, AEE obtained by means of the RT3; AEE_ref_, total energy expenditure measured using the doubly labelled water method during days 1–15 minus BMR measured using indirect calorimetry; AEE_5dresult_, total energy expenditure, measured using the doubly labelled water method during days 1–5 minus BMR measured using indirect calorimetry.

* Mean value was significantly different from that for AEE_ref_ (*P* < 0·001).

† Mean value was significantly different from that for AEE_5dresult_ (*P* < 0·001).

### Bland–Altman evaluations

**Fig. 1.**
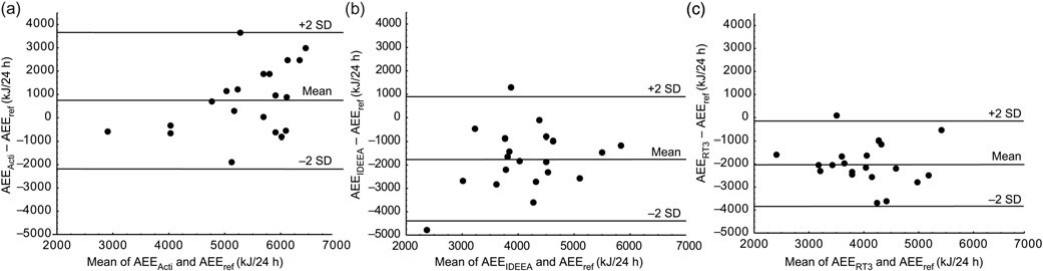
Bland–Altman plots comparing activity energy expenditure (AEE) assessed using monitors *v.* reference estimates. (a) AEE obtained using the Actiheart (AEE_Acti_) *v.* AEE measured using a combination of the doubly labelled water method and indirect calorimetry (AEE_ref_). AEE_Acti_ – AEE_ref_ was 740 kJ per 24 h (2 sd 2940). The regression equation was *y* = 0·68*x* – 2923; *r* 0·42 (*P* > 0·05). (b) AEE obtained using the IDEEA (AEE_IDEEA_) *v.* AEE_ref_. AEE_IDEEA_ – AEE_ref_ was –1750 kJ per 24 h (2 sd 2650). The regression equation was *y* = 0·46*x* – 3645; *r* 0·28 (*P* > 0·05). (c) AEE obtained using the RT3 (AEE_RT3_) *v.* AEE_ref_. AEE_RT3_ – AEE_ref_ was –2010 kJ per 24 h (2 sd 1820). The regression equation was *y* = –0·14*x* – 1462; *r* 0·11 (*P* > 0·05).

Fig. 1 shows Bland–Altman plots for AEE_Acti_ (a), AEE_IDEEA_ (b) and AEE_RT3_ (c). The mean differences, in kJ per 24 h, *v.* AEE_ref_ were 740 (Actiheart), –1750 (IDEEA) and –2010 (RT3). The limits of agreement (in kJ per 24 h) were wide for all monitors, i.e. 2940 (Actiheart), 2650 (IDEEA) and 1820 (RT3). The mean differences for results were: AEE_Acti_ – AEE_ref_: 11·5 (sd 25·2) kJ per 24 h per kg; AEE_IDEEA_ – AEE_ref_: –26·5 (sd 23·0) kJ per 24 h per kg; and AEE_RT3_ – AEE_ref_: –30·5 (sd 15·2) kJ per 24 h per kg. No significant linear relationships could be identified when the difference between estimates obtained using any of the monitors and the corresponding reference estimate was regressed on the average of the same two estimates. This was true when results were expressed in kJ per 24 h as well as in kJ per 24 h per kg. As a consequence of these results, the following evaluations were considered necessary.

### Comparison of means

A statistical comparison of AEE_Acti_, AEE_IDEEA_ and AEE_RT3_ *v.* AEE_ref_ is shown in Table 2. When expressed in kJ per 24 h, average AEE_Acti_ was 14·7 % higher than average AEE_ref_, but the difference was not statistically significant. AEE_RT3_ and AEE_IDEEA_ underestimated (*P* < 0·001) AEE_ref_ by 40 and 35 %, respectively. Furthermore, as also shown in Table 2, similar results were obtained when AEE was expressed per kg body weight (kJ per 24 h per kg).

### Correlations

When AEE_ref_ was correlated with AEE_Acti_, AEE_IDEEA_ or AEE_RT3_ no significant relationships were obtained, neither for values expressed in kJ per 24 h, nor for values expressed in kJ per 24 h per kg. Furthermore, AEE_IDEEA_ – AEE_ref_, AEE_RT3_ – AEE_ref_ and AEE_Acti_ – AEE_ref_, in kJ per 24 h or in kJ per 24 h per kg, were not correlated with each other.

### Comparison of variances

The variance for AEE_RT3_ was significantly lower than that for AEE_Acti_ when values were expressed in kJ per 24 h (*P* = 0·006) or in kJ per 24 h per kg (*P* < 0·001). The variance of AEE_IDEEA_ was not significantly different from the variances of AEE_RT3_ or AEE_Acti_ when values obtained during 5 d were compared.

### Classification capacity

**Fig. 2.**
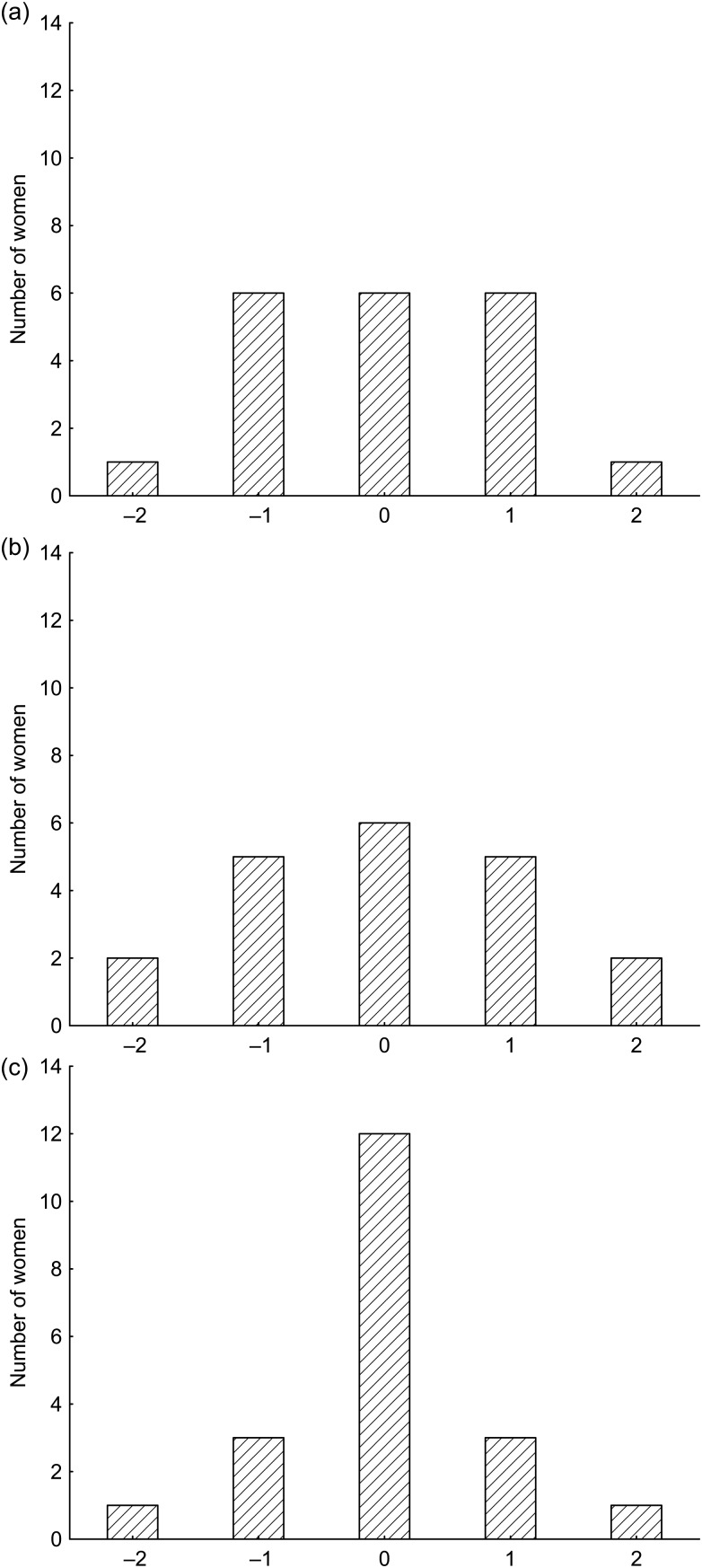
Capacity of activity monitors to classify activity energy expenditure (AEE). Number of women classified in the same (0), in the next higher (+1) or lower (–1), in the second next higher (+2) or lower (–2) group as compared with groups obtained when the classification was based on AEE assessed using a combination of the doubly labelled water method and indirect calorimetry (AEE_ref_). (a) AEE obtained using the Actiheart (AEE_Acti_). (b) AEE obtained using the IDEEA (AEE_IDEEA_). (c) AEE obtained using the RT3 (AEE_RT3_).

Fig. 2 shows the capacity of the Actiheart, the IDEEA and the RT3 to classify estimates of AEE when compared with AEE_ref_. The Actiheart and the IDEEA both classified only six women (30 %) correctly while the corresponding figure for RT3 was twelve (60 %). Correspondingly, when classification was based on Counts_RT3_ fourteen women (70 %) were correctly classified while Counts_IDEEA_ or Counts_Acti_ classified only six women (30 %) correctly. When classification was based on mean HRaR, six women (30 %) were correctly classified. Similar classification capacity results were obtained when AEE_ref_, AEE_Acti_, AEE_IDEEA_ and AEE_RT3_ were expressed in kJ per 24 h per kg (data not shown).

## Discussion

As indicated above, estimates of AEE are often corrected by deducting a value for DIT equal to 10 % of TEE. However, this is based on studies showing that DIT varies between 5 and 15 % of TEE^(^^34^^)^ and therefore AEE values with this correction cannot be considered to represent accurate estimates of AEE. On the other hand, for example, although our estimates of AEE_Acti_ include some DIT since calibrations were conducted with subjects in the fed state, it cannot be stated with assurance that the amount of DIT included is an accurate estimate of DIT during 24 h. Thus AEE assessed by monitors is never quite comparable with AEE assessed using reference methods. However, for the reasons provided in the paragraph below, we do not consider that this problem has affected the validity of the present results.

We calculated AEE as TEE – BMR with no correction for DIT since this was the most appropriate way to compare results obtained by the three monitors. An alternative possibility, however, is to base the comparison on PAL values. Since PAL is calculated as TEE/BMR it provides an estimate of physical activity that is independent of body weight. Body weight is associated with TEE and thus with the magnitude of DIT as well as with BMR. Since PAL is less affected than AEE by variations in DIT, PAL values are commonly calculated without any correction for DIT. We provide results for PAL assessed using the Actiheart, the RT3 and the IDEEA *v.* PAL_ref_ in supplementary material (online Supplementary Tables S1, online Supplementary Table S2; and online Supplementary Fig. S1). These two tables, S1 and S2, and Fig. S1 show that the present results are similar and our conclusions identical when using PAL rather than AEE. Therefore we consider that our findings regarding the capacity of the three monitors to assess energy expenditure in response to physical activity represent valid results.

All twenty women carried the RT3 during the complete 14 d period while fourteen women carried Actiheart and IDEEA, for 14 and 5 complete days, respectively. The present results and conclusions were the same when calculations were based on these women only.

In our women, average AEE_Acti_ was nearly 15 % higher than average AEE_ref_ and this difference was not significant. This raises the question if the present study was too small to identify a significant overestimation in AEE by the Actiheart. As mentioned above, we expected Actiheart to produce average estimates of AEE in agreement with reference values since the calculations are based on a calibration procedure. Heart rate recording is an important component of this procedure and Westerterp^(^^6^^)^ compiled results from eleven different studies and concluded that none of them identified any significant difference at the group level between TEE assessed by means of heart rate recording *v.* doubly labelled water. Validation studies of Actiheart using reference methods in Cameroon adults^(^^15^^)^, in US children^(^^35^^)^ and in French adult men^(^^21^^)^ or using a combination of a heart rate recorder and an accelerometer^(^^36^^,^^37^^)^ indicate that, when compared with criterion methods at the group level, such estimates of AEE produce non-significant overestimates^(^^35^^–^^37^^)^ ranging from +0·1 to +26·8 % or non-significant underestimates^(^^15^^,^^21^^)^, ranging from –7·9 to –9·1 %. It should be noted, however, that these results^(^^15^^,^^21^^,^^35^^)^ may not be quite comparable since there are minor variations in experimental conditions regarding how reference AEE is calculated, how Actiheart is applied to assess energy expenditure, and how the recorded information is used to calculate AEE. Nevertheless, the facts above provide substantial evidence for the statement that Actiheart is able to assess the average AEE of groups and our findings can be reconciled with this statement. This is important considering the need for methods to accurately assess the average energy expenditure of groups when evaluating the validity of dietary intake data^(^^23^^)^. For such validation simpler methods, such as physical activity questionnaires, may be considered. However, such methods often have poor criterion validity at the group level^(^^5^^)^ and may be influenced by subjective factors. Therefore, although far from perfect, the Actiheart must still be regarded as superior to questionnaires for this application.

Contrary to previous findings^(^^15^^,^^21^^,^^35^^)^, AEE_Acti_ and AEE_ref_ were not correlated in the present study, possibly because our population covered a more narrow range of AEE than was done by previous studies^(^^15^^,^^21^^,^^35^^)^. The limits of agreement for AEE_Acti_ were wide in the present study with 2 sd being equivalent to 50 % of AEE_ref_. The present results for AEE_Acti_ – AEE_ref_ in kJ per 24 h can be compared with the corresponding results, 4 (sd 824) kJ per 24 h, as reported by Butte *et al.*^(^^35^^)^. Their sd value, 824, is equivalent to about 32 % of average reference AEE of the subjects in their study^(^^35^^)^ while the corresponding value for our subjects is about 29 %. The present results for AEE_Acti_ – AEE_ref_ (mean 11·5 (sd 25·2) kJ per 24 h per kg) can be compared with the corresponding results by Assah *et al.*^(^^15^^)^ (mean –5·4 (sd 29·0) kJ per 24 h per kg) and by Villars *et al.*^(^^21^^)^ (mean –4·6 (sd 13·1) kJ per 24 h per kg). Villars *et al.*^(^^21^^)^ found the capacity of Actiheart to assess AEE at the individual level to be better than that observed in either the present study or in the study by Assah *et al.*^(^^15^^)^. Again, variations in experimental conditions may be responsible for these discrepancies. Thus, in the study by Assah *et al.*^(^^15^^)^ calibration was conducted using the so-called step-test rather than the individual calibrations that we used and which Villars *et al.*^(^^21^^)^ found to produce the most accurate results. The studies by Assah *et al.*^(^^15^^)^ and by Villars *et al.*^(^^21^^)^ recorded data throughout 24 h periods, while our subjects did not carry Actiheart during sleep. The latter discrepancy is not likely to be important, however, since AEE during sleep is close to zero. Regarding the different results found in the present study and in the study by Villars *et al.*^(^^21^^)^, it may be relevant that the equations in the Actiheart software were, as far as we have been able to assess, primarily developed in men^(^^38^^,^^39^^)^ and may have been inappropriate for our women. Brage *et al.*^(^^40^^)^ reported higher noise rates in women than in men when using Actiheart, suggesting that this sex difference is due to signal attenuation caused by more subcutaneous fat in women than in men. Additional work may be needed to make the Actiheart procedure more suitable for women.

The present results show that the IDEEA device underestimated AEE by approximately 35 %. This result is based on a comparison with AEE_ref_. Comparing average AEE_IDEEA_ with average AEE_5dresult_ produced very similar results. A Bland–Altman evaluation similar to that shown in Fig. 1(b), but based on AEE_5dresult_ rather than on AEE_ref_, showed wider limits of agreement, 2 sd being 3027 rather than 2650 kJ per 24 h. An important explanation for this observed increase may be the imprecision in AEE_5dresult_. Our observation that the IDEEA underestimated AEE is in contrast to a previous evaluation of a similar device by Whybrow *et al.*^(^^20^^)^ who reported an overestimation of AEE by 48 %. Furthermore, the limits of agreement obtained in the Bland–Altman evaluation of AEE were smaller in the present study where ± 2 sd ranged between –4·4 and 0·9 MJ per 24 h. The corresponding range in the study by Whybrow *et al.*^(^^20^^)^ was from –2·7 to 4·5 MJ per 24 h. However, it is difficult to compare their evaluation with ours since they used a modified version of the IDEEA with eight sensors instead of five. Furthermore, their protocol was different and we do not know to what extent their calculations were different from ours. One obvious explanation for the underestimation of AEE by means of the IDEEA in the present study is the use of energy expenditure when lying down as an estimate of resting energy metabolism as recommended by the manufacturer. Using our measured BMR to calculate AEE_IDEEA_ reduced the underestimation to 1 %. However, when we calculated AEE_IDEEA_ in this way, neither the correlation with AEE_ref_ nor the classification capacity was improved. Several reports show that the IDEEA can classify activities accurately^(^^41^^–^^43^^)^ and therefore the poor results are probably due to errors introduced when calculating energy expenditure. Unfortunately, the details regarding this calculation are unknown to us, making it difficult to suggest explanations for our observations. However, a possible explanation for the inaccuracy at the individual level may be that IDEEA uses MET values to calculate energy expenditure and such values are known to vary between individuals^(^^31^^)^. It may also be relevant to note that energy expenditure due to fidgeting is unlikely to be included in the IDEEA estimates^(^^13^^)^. Levine *et al.*^(^^44^^)^ reported that energy expenditure during sitting and standing while fidgeting could be 46 % and 69 % higher than when sitting and standing motionless, respectively. We conclude that the IDEEA results showed wide limits of agreement indicating poor accuracy at the individual level.

Average AEE_RT3_ was 40 % lower than average AEE_ref_, and the RT3 underestimated AEE in all women except one. Three studies have evaluated the RT3 against reference methods^(^^16^^,^^21^^,^^45^^)^ and all showed lower average AEE values *v.* average reference AEE, i.e. –15 %^(^^16^^)^, –17 %^(^^45^^)^ and –33 %^(^^21^^)^. Regarding results at the individual level, our findings are in agreement with data for lean men as reported by Villar *et al.*^(^^21^^)^ who found that the sd of the difference between AEE_ref_ and AEE_RT3_ varied between 14·2 and 17·7 when results were expressed in kJ per 24 h per kg. The corresponding figure in the present study was 15·2 kJ per 24 h per kg. Other relevant findings were presented by Westerterp and colleagues^(^^17^^)^, who have validated different versions of a three-axial accelerometer, Tracmor, for more than a decade. Acceleration measured using Tracmor was found to explain the largest variation in TEE when compared with other accelerometers^(^^17^^,^^18^^)^. In recent publications, average PAL, assessed by means of Tracmor, was shown to agree with PAL measured using reference methods^(^^46^^)^ and average AEE, assessed by means of the three-axial accelerometer GENEA, was in good agreement with reference AEE^(^^18^^)^. Thus, the problem with underestimation possibly associated with multiaxial accelerometry may be overcome, but more studies are needed to confirm this.

Although the RT3 was found to underestimate average AEE_ref_, its performance at the individual level was good in comparison with the Actiheart. Thanks to the design of the present study where the monitors were evaluated in the same women, we were able to demonstrate that the RT3 was superior to the Actiheart regarding its ability to provide relevant estimates of AEE for individuals. Bonomi *et al.*^(^^46^^)^ showed that Tracmor also is very satisfactory at the individual level. Furthermore, it should be noted that the RT3 is a very user-friendly device since it does not require calibration procedures demanding extra resources. Some of our women complained that the electrodes needed to attach Actiheart caused itching. An advantage with RT3 is certainly that it does not require any such electrodes. Furthermore, good results can apparently be obtained at the individual level by means of RT3 even if subjects only wear the monitors during the daytime, which is a considerable advantage from a practical point of view.

In the present study we used equations provided by the manufacturers to calculate AEE from raw output data (i.e. heart rate and counts). However, when classification capacity was calculated using either raw output data or the corresponding AEE values, similar results were obtained. Obviously, the equations used did not change the interindividual variation in the investigated variable as assessed by any of the three devices.

A strength of the present study is that we measured TEE and BMR using methods with high accuracy and precision while a limitation is the small sample size, which limits generalisability. Furthermore, the present results are limited to women of reproductive age. However, our women covered a wide range of BMI values and their average PAL was similar to that of European and American women aged 20–45 years^(^^47^^)^. Furthermore, the range in PAL values observed for our women (1·65–2·11) covers such values for many Western women^(^^48^^)^. Thus the present results are likely to be relevant for many subjects in the Western world. However, this does not mean that the present results are necessarily applicable in such populations since many subjects, also in the Western world, may have a different pattern of physical activity.

The present results and the discussion above demonstrate the complexity involved when assessing human physical activity. Obviously, evaluations at the group as well as at the individual level are required to assess the full potential of any procedure intended to assess energy expended in response to physical activity during free-living conditions. The three devices studied in the present paper have strengths and weaknesses and are therefore likely to be useful in different situations. Thus, Actiheart may well be useful when the average AEE of a population is of interest, for example when validating assessments of energy intake for a group of subjects. However, the inaccuracy at the individual level is a concern. In studies where differences between individuals are of interest, for example, when physical activity is linked to the risk for a disease, a multiaxial accelerometer may be preferable. Furthermore, when evaluating different devices it is important to consider the physical activity of the study population. It is much easier to identify differences in physical activity between individuals in a population with a large variation in physical activity. However, in studies of the health effects of physical activity, rather small differences between individuals may be of interest. We recommend that any method for assessing physical activity should be evaluated in a population comparable with that in which it is to be applied, and furthermore that the evaluation procedure should be appropriate for the specific research question of the study. If there is a choice between methods, it may be advantageous if each method can be tested in representative subjects before starting the main study.

We evaluated the capacity of three devices to assess AEE at the group and at the individual level. The study was conducted in one group of women, which contributed to revealing the strengths and weaknesses of the devices. The results can be reconciled with previous results indicating that Actiheart has the capacity to assess the physical activity of groups although its inaccuracy at the individual level is a concern. The present results demonstrated limitations of the IDEEA system, and the potential of the three-axial accelerometer RT3 to study physical activity of individuals. Our findings highlight the need to apply appropriate procedures when studying interactions between physical activity, energy intake and health.

## Supplementary Material

Supplementary Material Supplementary information supplied by authors.

## Supplementary material

To view supplementary material for this article, please visit http://dx.doi.org/10.1017/jns.2013.18

## References

[1] World Health Organization (2003) Diet, Nutrition and the Prevention of Chronic Diseases. Joint WHO/FAO Expert Consultation. WHO Technical Report Series no. 916. Geneva: WHO 12768890

[2] Ekelund U, Besson H, Luan J, (2011) Physical activity and gain in abdominal adiposity and body weight: prospective cohort study in 288,498 men and women. Am J Clin Nutr 93, 826–835 21346093 10.3945/ajcn.110.006593

[3] Woodcock J, Franco OH, Orsini N, (2011) Non-vigorous physical activity and all-cause mortality: systematic review and meta-analysis of cohort studies. Int J Epidemiol 40, 121–138 20630992 10.1093/ije/dyq104

[4] Caspersen CJ, Powell KE & Christenson GM (1985) Physical activity, exercise, and physical fitness: definitions and distinctions for health-related research. Public Health Rep 100, 126–131 3920711 PMC1424733

[5] Neilson HK, Robson PJ, Friedenreich CM, (2008) Estimating activity energy expenditure: how valid are physical activity questionnaires? Am J Clin Nutr 87, 279–291 18258615 10.1093/ajcn/87.2.279

[6] Westerterp KR (2009) Assessment of physical activity: a critical appraisal. Eur J Appl Physiol 105, 823–828 19205725 10.1007/s00421-009-1000-2

[7] Bonomi AG & Westerterp KR (2012) Advances in physical activity monitoring and lifestyle interventions in obesity: a review. Int J Obes 36, 167–177 10.1038/ijo.2011.99 21587199

[8] Melzer K, Schutz Y, Boulvain M, (2009) Pregnancy-related changes in activity energy expenditure and resting metabolic rate in Switzerland. Eur J Clin Nutr 63, 1185–1191 19550432 10.1038/ejcn.2009.49

[9] Turner JE, Markovitch D, Betts JA, (2010) Nonprescribed physical activity energy expenditure is maintained with structured exercise and implicates a compensatory increase in energy intake. Am J Clin Nutr 92, 1009–1016 20826629 10.3945/ajcn.2010.29471

[10] Wynne K, Park AJ, Small CJ, (2006) Oxyntomodulin increases energy expenditure in addition to decreasing energy intake in overweight and obese humans: a randomised controlled trial. Int J Obes 30, 1729–1736 10.1038/sj.ijo.0803344 16619056

[11] CamNtech Ltd (2012) CamNtech web site. http://www.camntech.com (accessed 24 April 2013).

[12] Plasqui G, Bonomi AG & Westerterp KR (2013) Daily physical activity assessment with accelerometers: new insights and validation studies. Obes Rev 14, 451–462 23398786 10.1111/obr.12021

[13] Zhang K, Pi-Sunyer FX & Boozer CN (2004) Improving energy expenditure estimation for physical activity. Med Sci Sports Exerc 36, 883–889 15126725 10.1249/01.mss.0000126585.40962.22

[14] Zhang K, Werner P, Sun M, (2003) Measurement of human daily physical activity. Obes Res 11, 33–40 12529483 10.1038/oby.2003.7

[15] Assah FK, Ekelund U, Brage S, (2011) Accuracy and validity of a combined heart rate and motion sensor for the measurement of free-living physical activity energy expenditure in adults in Cameroon. Int J Epidemiol 40, 112–120 20529884 10.1093/ije/dyq098

[16] Maddison R, Jiang Y, Hoorn SV, (2009) Estimating energy expenditure with the RT3 triaxial accelerometer. Res Q Exerc Sport 80, 249–256 19650390 10.1080/02701367.2009.10599559

[17] Plasqui G & Westerterp KR (2007) Physical activity assessment with accelerometers: an evaluation against doubly labeled water. Obesity 15, 2371–2379 17925461 10.1038/oby.2007.281

[18] van Hees VT, Renström F, Wright A, (2011) Estimation of daily energy expenditure in pregnant and non-pregnant women using a wrist-worn tri-axial accelerometer. PloS ONE 6, e22922. 10.1371/journal.pone.0022922 PMC3146494 21829556

[19] Van Remoortel H, Giavedoni S, Raste Y, (2012) Validity of activity monitors in health and chronic disease: a systematic review. Int J Behav Nutr Phys Act 9, 84. 10.1186/1479-5868-9-84 PMC3464146 22776399

[20] Whybrow S, Ritz P, Horgan GW, (2013) An evaluation of the IDEEA^TM^ activity monitor for estimating energy expenditure. Br J Nutr 109, 173–183 22464547 10.1017/S0007114512000645

[21] Villars C, Bergouignan A, Dugas J, (2012) Validity of combining heart rate and uniaxial acceleration to measure free-living physical activity energy expenditure in young men. J Appl Physiol 113, 1763–1771 23019315 10.1152/japplphysiol.01413.2011

[22] Bland JM & Altman DG (1986) Statistical methods for assessing agreement between two methods of clinical measurement. Lancet i, 307–310 2868172

[23] Hill RJ & Davies PS (2001) The validity of self-reported energy intake as determined using the doubly labelled water technique. Br J Nutr 85, 415–430 11348556 10.1079/bjn2000281

[24] Bexelius C, Löf M, Sandin S, (2010) Measures of physical activity using cell phones: validation using criterion methods. J Med Internet Res 12, e2. 10.2196/jmir.1298 PMC2821583 20118036

[25] Löf M (2011) Physical activity pattern and activity energy expenditure in healthy pregnant and non-pregnant Swedish women. Eur J Clin Nutr 65, 1295–1301 21792212 10.1038/ejcn.2011.129

[26] Weir JB (1949) New methods for calculating metabolic rate with special reference to protein metabolism. J Physiol 109, 1–9 15394301 10.1113/jphysiol.1949.sp004363 PMC1392602

[27] Lof M, Hannestad U & Forsum E (2003) Comparison of commonly used procedures, including the doubly-labelled water technique, in the estimation of total energy expenditure of women with special reference to the significance of body fatness. Br J Nutr 90, 961–968 14667189 10.1079/bjn2003975

[28] Coward WA (1988) Stable isotopic methods for measuring energy expenditure. The doubly-labelled-water (^2^H_2_^18^O) method: principles and practice. Proc Nutr Soc 47, 209–218 3076004 10.1079/pns19880037

[29] Black AE, Prentice AM & Coward WA (1986) Use of food quotients to predict respiratory quotients for the doubly-labelled water method of measuring energy expenditure. Hum Nutr Clin Nutr 40, 381–391 3771290

[30] International Atomic Energy Agency (2009) Assessment of Body Composition and Total Energy Expenditure in Human Using Stable Isotope Techniques. Human Health Series no. 3. Vienna: International Atomic Energy Agency

[31] Ainsworth BE, Haskell WL, Whitt MC, (2000) Compendium of physical activities: an update of activity codes and MET intensities. Med Sci Sports Exerc 32, S498–S504 10993420 10.1097/00005768-200009001-00009

[32] Schoeller DA (1983) Energy expenditure from doubly labeled water: some fundamental considerations in humans. Am J Clin Nutr 38, 999–1005 6650456 10.1093/ajcn/38.6.999

[33] Pitman E (1939) A note on normal correlation. Biometrika 31, 9–12

[34] Westerterp KR (2004) Diet induced thermogenesis. Nutr Metab (Lond) 1, 5. 10.1186/1743-7075-1-5 PMC524030 15507147

[35] Butte NF, Wong WW, Adolph AL, (2010) Validation of cross-sectional time series and multivariate adaptive regression splines models for the prediction of energy expenditure in children and adolescents using doubly labeled water. J Nutr 140, 1516–1523 20573939 10.3945/jn.109.120162 PMC2903304

[36] Hustvedt BE, Christophersen A, Johnsen LR, (2004) Description and validation of the ActiReg: a novel instrument to measure physical activity and energy expenditure. Br J Nutr 92, 1001–1008 15613263 10.1079/bjn20041272

[37] Patrik Johansson H, Rossander-Hulthen L, Slinde F, (2006) Accelerometry combined with heart rate telemetry in the assessment of total energy expenditure. Br J Nutr 95, 631–639 16512950 10.1079/bjn20051527

[38] Brage S, Brage N, Franks PW, (2005) Reliability and validity of the combined heart rate and movement sensor Actiheart. Eur J Clin Nutr 59, 561–570 15714212 10.1038/sj.ejcn.1602118

[39] Brage S, Brage N, Franks PW, (2004) Branched equation modeling of simultaneous accelerometry and heart rate monitoring improves estimate of directly measured physical activity energy expenditure. J Appl Physiol 96, 343–351 12972441 10.1152/japplphysiol.00703.2003

[40] Brage S, Brage N, Ekelund U, (2006) Effect of combined movement and heart rate monitor placement on physical activity estimates during treadmill locomotion and free-living. Eur J Appl Physiol 96, 517–524 16344938 10.1007/s00421-005-0112-6

[41] Marsh AP, Vance RM, Frederick TL, (2007) Objective assessment of activity in older adults at risk for mobility disability. Med Sci Sports Exerc 39, 1020–1026 17545894 10.1249/mss.0b013e3180423ac3

[42] Saremi K, Marehbian J, Yan X, (2006) Reliability and validity of bilateral thigh and foot accelerometry measures of walking in healthy and hemiparetic subjects. Neurorehabil Neural Repair 20, 297–305 16679506 10.1177/1545968306287171

[43] Welk GJ, McClain JJ, Eisenmann JC, (2007) Field validation of the MTI Actigraph and BodyMedia armband monitor using the IDEEA monitor. Obesity 15, 918–928 17426327 10.1038/oby.2007.624

[44] Levine JA, Schleusner SJ & Jensen MD (2000) Energy expenditure of nonexercise activity. Am J Clin Nutr 72, 1451–1454 11101470 10.1093/ajcn/72.6.1451

[45] Jacobi D, Perrin AE, Grosman N, (2007) Physical activity-related energy expenditure with the RT3 and TriTrac accelerometers in overweight adults. Obesity 15, 950–956 17426330 10.1038/oby.2007.605

[46] Bonomi AG, Plasqui G, Goris AH, (2009) Improving assessment of daily energy expenditure by identifying types of physical activity with a single accelerometer. J Appl Physiol 107, 655–661 19556460 10.1152/japplphysiol.00150.2009

[47] Food and Nutrition Board & Institute of Medicine (2005) Dietary Reference Intakes for Energy, Carbohydrate, Fiber, Fat, Fatty Acids, Cholesterol, Protein and Amino Acids (Macronutrients). Washington, DC: The National Academies Press

[48] Scientific Advisory Committee on Nutrition (SACN) (2011) Dietary reference values for energy. http://www.sacn.gov.uk/ (accessed 24 April 2013).

