# Supplementary material for: Evaluations of Actiheart, IDEEA® and RT3 monitors for estimating activity energy expenditure in free-living women
Source: J Nutr Sci. 2013 Sep 6;2:e31. doi: 10.1017/jns.2013.18 (PMC4153312; doi:10.1017/jns.2013.18)
Supplement: Supplementary Material — Supplementary information supplied by authors. [file S2048679013000189sup001.doc]

**Table S1.** Mean and standard deviation (SD) for physical activity levels (PAL) assessed by means of the Actiheart, the IDEEA, the RT3 and reference methods (n= 20).

|  | Mean | SD |
| --- | --- | --- |
| PAL_Acti_* | 1·97 | 0·25 |
| PAL_RT3_^†^ | 1·51^ǁ^ | 0·13 |
| PAL_IDEEA_^‡^ | 1·44^ǁ^ | 0·18 |
| PAL_ref_^§^ | 1·85 | 0·13 |
|  |  |  |

PAL_Acti_, PAL assessed using Actiheart; PAL_RT3_, PAL assessed using RT3; PAL_IDEEA_, PAL assessed using IDEEA; PAL_ref_, PAL assessed using reference methods

^*^Calculated as [activity energy expenditure assessed by means of Actiheart (kJ/24h) as described in the method section plus basal metabolic rate measured using indirect calorimetry (kJ/24h)] divided by basal metabolic rate measured using indirect calorimetry (kJ/24h)

^†^ Calculated as [activity expenditure (kJ/24h) assessed by means of the RT3 as described in the method section plus basal metabolic rate measured using indirect calorimetry (kJ/24h)] divided by basal metabolic rate measured using indirect calorimetry (kJ/24h).

^‡^ Calculated as total energy expenditure (kJ/24h) assessed by means of the IDEEA as described in the method section divided by an estimate of resting energy expenditure (kJ/24h). For these calculations, estimates of resting energy expenditure provided by means of the IDEEA were used (as described in the method section).

^§^ Calculated as total energy expenditure measured using the doubly labeled water method (kJ/24h) divided by basal metabolic rate measured using indirect calorimetry (kJ/24h)

^ǁ^Significantly different from PAL_ref_ (P<0·001)

**Table S2.** Mean and standard deviation (SD) for the difference between physical activity levels (PAL) assessed by means of the Actiheart, the IDEEA, the RT3, respectively, and PAL assessed using reference methods (n= 20).

|  | Mean | SD |
| --- | --- | --- |
| PAL_Acti_ -PAL_ref_ | 0·12 | 0·23 |
| PAL_RT3_-PAL_ref_ | -0·34 | 0·15 |
| PAL_IDEEA_-PAL_ref_ | -0·41 | 0·24 |

PAL_Acti_, PAL assessed using Actiheart; PAL_RT3_, PAL assessed using RT3; PAL_IDEEA_, PAL assessed using IDEEA; PAL_ref_, PAL assessed using reference methods

Comment to Table S2: PAL_Acti_, PAL_RT3_, PAL_IDEEA_ and PAL_ref_ were calculated as described in Table S1.

**Figure S1.** **The capacity of activity monitors to classify physical activity level (PAL).**

The figure shows the number of women classified in the same (0), in the next higher (+1) or lower (-1), in the second next higher (+2) or lower (-2) group as compared to groups obtained when the classification was based on physical activity level (PAL) assessed using a combination of the doubly labeled water method and indirect calorimetry (PAL_ref_).

a) PAL obtained using the Actiheart (PAL_Acti_)

b) PAL obtained using the IDEEA (PAL_IDEEA)_

c) PAL obtained using the RT3 (PAL_RT3)_

Comment to figure S1: PAL_ref_, PAL_Acti_, PAL_IDEEA_ and PAL_RT3_ in figure S1 are calculated as described in Table S1. The Actiheart and IDEEA classified 7 (35 %) and 5(25 %) women correctly, respectively, while the corresponding figure for the RT3 was 12 women (60 %).


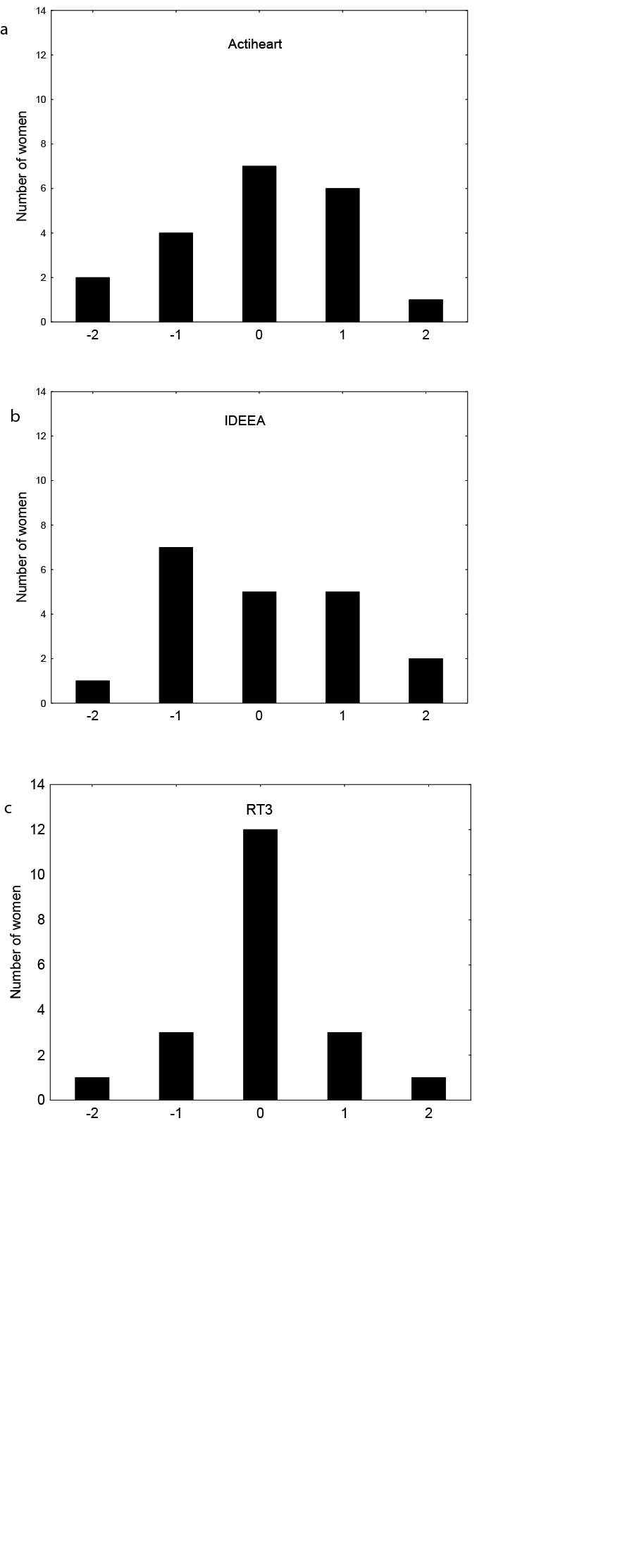


Figure S1
